# A Digital Mental Health Solution to Improve Social, Emotional, and Learning Skills for Youth: Protocol for an Efficacy and Usability Study

**DOI:** 10.2196/59372

**Published:** 2024-12-19

**Authors:** Kayla V Taylor, Laurent Garchitorena, Carolina Scaramutti-Gladfelter, Mykayla Wyrick, Katherine B Grill, Azizi A Seixas

**Affiliations:** 1 Department of Informatics and Health Data Science University of Miami Miller School of Medicine Miami, FL United States; 2 Department of Psychiatry and Behavioral Science University of Miami Miller School of Medicine Miami, FL United States; 3 Neolth Company Los Angeles, CA United States

**Keywords:** mental health, digital health, mHealth, usability, pilot study, United States, mental health crisis, Science Technology Engineering Math and Social and Emotional Learning, STEMSEL, efficacy, well-being, barriers, facilitators, resources, youth, adolescents, teenagers, students, feasibility, adoption, evidence-based, intervention, anxiety, depression, Neolth, digital app

## Abstract

**Background:**

The COVID-19 pandemic has exposed a devastating youth mental health crisis in the United States, characterized by an all-time high prevalence of youth mental illness. This crisis is exacerbated by limited access to mental health services and the reduction of mental health support in schools. Mobile health platforms offer a promising avenue for delivering tailored and on-demand mental health care.

**Objective:**

To address the lack of youth mental health services, we created the Science Technology Engineering Math and Social and Emotional Learning (STEMSEL) study. Our aim was to investigate the efficacy of a digital mental health intervention, Neolth, in enhancing social and emotional well-being, reducing academic stress, and increasing mental health literacy and life skills among adolescents.

**Methods:**

The STEMSEL study will involve the implementation and evaluation of Neolth across 4 distinct phases. In phase 1, a comprehensive needs assessment will be conducted across 3 diverse schools, each using a range of teaching methods, including in-person, digital, and hybrid modalities. Following this, in phase 2, school administrators and teachers undergo intensive training sessions on Neolth’s functionalities and intervention processes as well as understand barriers and facilitators of implementing a digital mental health program at their respective schools. Phase 3 involves recruiting middle and high school students aged 11-18 years from the participating schools, with parental consent and student assent obtained, to access Neolth. Students will then be prompted to complete an intake questionnaire, enabling the customization of available modules to address their specific needs. Finally, phase 4 will include a year-long pre- and posttest pilot study to rigorously evaluate the usability and effectiveness of Neolth in addressing the mental health concerns of students across the selected schools.

**Results:**

Phase 1 was successfully completed in August 2022, revealing significant deficits in mental health resources within the participating schools. The needs assessment identified critical gaps in available mental health support services. We are currently recruiting a diverse group of middle and high school students to participate in the study. The study’s completion is scheduled for 2024, with data expected to provide insights into the real-world use of Neolth among the adolescent population. It is designed to deliver findings regarding the intervention’s efficacy in addressing the mental health needs of students.

**Conclusions:**

The STEMSEL study plays a crucial role in assessing the feasibility and adoption of digital mental health interventions within the school-aged youth population in the United States. The findings generated from this study have the potential to dismantle obstacles to accessing mental health assistance and broaden the availability of care through evidence-based strategies.

**International Registered Report Identifier (IRRID):**

DERR1-10.2196/59372

## Introduction

### Background and Rationale

The burgeoning crisis of youth mental health underscores an urgent need for effective interventions, as barriers to accessing mental health care persist among this vulnerable demographic group. The school years represent a critical period in a young person’s life, marked by profound physical, emotional, and social changes, thus heightening their susceptibility to mental health issues [1]. The COVID-19 pandemic has intensified concerns about the rise in youth mental health challenges. The Centers for Disease Control and Prevention’s “Youth Risk Behavior Surveillance Data Summary & Trends Report: 2013-2023” declared the pandemic-related decline in adolescent mental health a national emergency. It reports nearly all indicators of poor mental health and suicidal thoughts and behaviors worsened from 2013 to 2023. Specifically, there were increases in the percentage of students who experienced persistent feelings of sadness or hopelessness, seriously considered attempting suicide, made a suicide plan, and attempted suicide. Around 32% of US teenagers are diagnosed with anxiety, and 12% with depression. More than 4 in 10 (42%) students reported feeling persistently sad or hopeless, signifying a significant prevalence of emotional distress among adolescents. Furthermore, nearly one-third (29%) of high school students reported experiencing poor mental health, reflecting a concerning trend that demands immediate attention and intervention. Additionally, more than 1 in 5 (22%) high school students seriously contemplated attempting suicide, highlighting the pervasive nature of suicidal ideation among this demographic. Even more alarming is the fact that 1 in 10 (10%) high school students reported attempting suicide, underscoring the urgent need for comprehensive strategies to address the mental well-being of our youth. These Centers for Disease Control and Prevention statistics paint a grim picture of the mental health landscape among youth and underscore the critical need for comprehensive mental health services. They serve as a sobering reminder of the vital role that public health interventions and initiatives must play in addressing the youth mental health crisis.

To confront this growing concern, innovative and evidence-based approaches are imperative, with a particular focus on enhancing access to mental health care, reducing stigma, and promoting social and emotional learning (SEL) within educational settings. However, barriers such as limited accessibility, affordability, and scalability of mental health services hinder access, particularly among resource-limited communities, exacerbating the youth mental health crisis [2]. Furthermore, stigma about mental illness is a formidable obstacle that discourages adolescents from seeking mental health support [3]. Stigma about mental health can lead to discrimination and isolation, exacerbating the challenges faced by young individuals. These multifaceted barriers highlight the urgent need to address these access disparities and reduce the stigma associated with mental health. To achieve this, innovative, creative, and adaptable solutions must be developed to increase access to and methods for promoting SEL, especially in a post–COVID-19 world. These solutions should not only target the various barriers often faced by adolescents but also align with their preferred methods of support. Safeguarding their well-being, nurturing socioemotional development, and ensuring access to mental health care are pivotal imperatives for their health and overall resilience.

The ever-evolving mobile health (mHealth) technology ecosystem and the widespread adoption of mobile devices present a unique opportunity to tackle the aforementioned challenges hindering youth access to quality mental health care. With nearly 6.4 billion smartphone mobile network subscriptions in 2022, and projections to reach 7.7 billion by 2028 [4], mHealth stands as a powerful platform for delivering on-demand mental health support and interventions to adolescents. Digital mental health services have demonstrated potential in engaging youth and have the advantage of circumventing the barriers that have long plagued traditional mental health care, including cost, availability, access, and personalized treatment [5]. In response, we created the Science Technology Engineering Math and Social and Emotional Learning (STEMSEL) study, aiming to explore the psychological, stress-related, emotional, and behavioral changes experienced by middle and high school students while evaluating whether a digital mental health solution, Neolth, can enhance social and emotional well-being, reduce academic stress, and increase mental health literacy and life skills among adolescents. The STEMSEL study holds the promise of leveraging digital and mobile mental health solutions to break down access barriers and reduce stigma, enabling young individuals to access quality mental health care anytime and anywhere.

### Theoretical Framework

The STEMSEL study draws upon the theoretical underpinnings of SEL and the transformative potential of digital technology in youth mental health care. SEL emphasizes vital life skills to navigate emotional and societal challenges, serving as protective factors for mental health. The study leverages digital tools to enhance access and effectiveness, aligning with policy priorities. Additionally, it builds upon the moderated online social therapy (MOST) framework, designed for young adults to address the unique needs of school-aged youth in stress, emotional well-being, mental health, and SEL. This theoretical foundation guides STEMSEL in bridging gaps in mental health care and promoting resilience among youth.

### Goal of SEL

The goal of SEL is to equip individuals, especially young students, with a set of crucial life skills that go beyond academic knowledge. SEL focuses on nurturing emotional intelligence, self-awareness, interpersonal skills, and resilience, among other essential attributes. By fostering these competencies, SEL aims to empower individuals to navigate the complex landscape of emotions, relationships, and societal interactions effectively. Importantly, these skills can serve as powerful protective factors against mental health issues. When students are equipped with strong SEL skills, they are better equipped to manage stress, cope with challenges, build healthy relationships, and maintain a positive sense of self. These competencies not only bolster their emotional well-being but also create a supportive environment that reduces the risk of mental health problems. Thus, the goal of SEL is not just academic success but also the promotion of mental health and overall well-being among students.

### Value of Digital Technology

The use of digital technology, particularly mHealth, holds significant promise in improving access, engagement, and treatment effectiveness in youth mental health care [6]. Extensive research spanning nearly 3 decades has demonstrated that internet-delivered treatments can greatly enhance accessibility and produce outcomes in engagement and effectiveness that are on par with traditional face-to-face psychological therapy [7,8]. Mental health system evaluations have called for the integration of digital interventions into service delivery, marking it as a national and international policy priority. For example, the mental health system in Australia enabled by digital technology enhanced accessibility and continuity of care [9].

However, challenges persist in user uptake, engagement, and adherence to digital interventions, particularly among young people, where rates of engagement in digital services for youth are lower compared to adults [10]. Additionally, there has been a historical global failure to effectively integrate digital interventions into real-world mental health services. To address these challenges and fully harness the potential of digital technology in youth mental health care, innovative solutions, accompanied by well-structured implementation and integration plans, are urgently needed [7,11-15].

The MOST framework, originally designed as a digital mental health platform for individuals aged 12-25 years, played a pivotal role in shaping the STEMSEL project. MOST provided the foundational framework for STEMSEL, offering a versatile structure for integrating digital interventions into established face-to-face youth mental health services. Stemming from MOST’s components, including evidence-based psychotherapeutic content, remote clinician and peer support, vocational guidance, and a peer-led digital community, STEMSEL adopted a flexible approach to address the diverse needs of youth navigating the complexities of adolescence.

The aims of the STEMSEL study are to evaluate the effectiveness of the Neolth digital mental health intervention in improving adolescents’ social and emotional well-being, reducing academic stress, and increasing mental health literacy and life skills. Specifically, the study seeks to assess the feasibility, usability, and real-world impact of integrating such digital interventions in diverse school settings, with the broader goal of addressing barriers to youth mental health care access, reducing stigma, and providing scalable solutions for resource-limited populations.

## Methods

### Study Design Overview

STEMSEL is a pre- and postdesign study that aims to increase SEL skills among Florida students in grades 6-12 using Neolth throughout the 2022-2024 academic years. As stated previously, Neolth is a mobile app that provides tier 1 resources for emotional awareness, coping skills, health education, and stigma reduction.

The study was designed to address the critical challenges in youth mental health care, leveraging the potential of digital and mobile mental health solutions. In the context of a rapidly evolving technological landscape, marked by the widespread adoption of mobile devices, the STEMSEL study seeks to harness the transformative power of mHealth interventions to enhance the accessibility, effectiveness, and acceptability of mental health support for school-aged youth.

The study is driven by four primary objectives (1) to assess whether Neolth effectively increases social and emotional wellness among students in grades 6-12, (2) to evaluate Neolth’s potential to decrease stress within an academic setting, (3) to examine whether Neolth contributes to improving mental health literacy and life skills among students, and (4) to investigate the rate of engagement and use of Neolth among students. These objectives encompass a comprehensive assessment of psychological, stress-related, emotional, behavioral, and sleep-related changes associated with the experiences of middle and high school students. Additionally, the study will investigate the usability and acceptance of digital mental health solutions, aiming to provide valuable insights into the transformative potential of such interventions in the context of youth mental health care.

### Study Procedures

#### Timeline

The STEMSEL study involves the implementation and evaluation of Neolth across 4 distinct phases. Phase 1 entailed conducting a comprehensive needs assessment across 3 participating schools, each with its own teaching methods and styles, ranging from in-person to hybrid models. This phase aimed to understand the specific mental health support requirements and challenges unique to each school setting. Subsequently, phase 2 focuses on administering a comprehensive training program to school administrators and teachers to familiarize them with Neolth’s functionalities, features, and intervention processes. The objective will be to equip educators with the necessary skills to effectively use the platform for supporting students’ mental health needs within their respective school environments. During phase 3, middle and high school students aged 11-18 years are recruited from the participating schools, with consent obtained from parents or legal guardians for students younger than 18 years of age, and assent secured from all participating students. During this phase, students gain access to the Neolth app, where they complete an intake questionnaire to customize available modules addressing their immediate needs. The final phase will involve the implementation of a pre- and posttest pilot study conducted over a 10-month period to assess the usability and effectiveness of Neolth in addressing the mental health needs of students within the STEMSEL study framework.

#### Participants

Participants in the STEMSEL initiative are recruited from 3 participating Florida schools. To be eligible for participation, individuals must meet specific inclusion criteria. First, participants must be aged 11 years or older. Second, they must be currently enrolled in 1 of the 3 participating schools. Third, they need to demonstrate English language proficiency to engage effectively with the intervention. Finally, parental consent is required for participation.

Conversely, certain exclusion criteria have been established to ensure the suitability of the sample. Individuals diagnosed with psychosis or those younger than 11 years of age are excluded from participation. Additionally, individuals who do not speak or understand English and those not enrolled in the participating institutions for the specified academic year are ineligible. Furthermore, individuals with a history of seizure, vertigo, significant vision or hearing impairment, significant motion sickness, epilepsy, or sensitivity to flashing light or motion, as well as those with injuries to the eyes, face, neck, or arms that would make the use of the app uncomfortable are also excluded from participation. These criteria are implemented to maintain consistency and ensure the eligibility of the sample size, which is targeted to exceed 60 participants.

#### Study Setting

Students will primarily engage in the research study through the Neolth mobile or web app. This app operates on mobile devices, tablets, or any desktop and serves as the primary platform for their participation. Guidance and encouragement to use this app will be provided by their teachers, who will play a pivotal role in facilitating their involvement. Students will be actively encouraged to use the Neolth app both within the school environment (1 time per week) and in their home settings, fostering comprehensive engagement in the study.

#### Recruitment Plan

**Figure 1:**
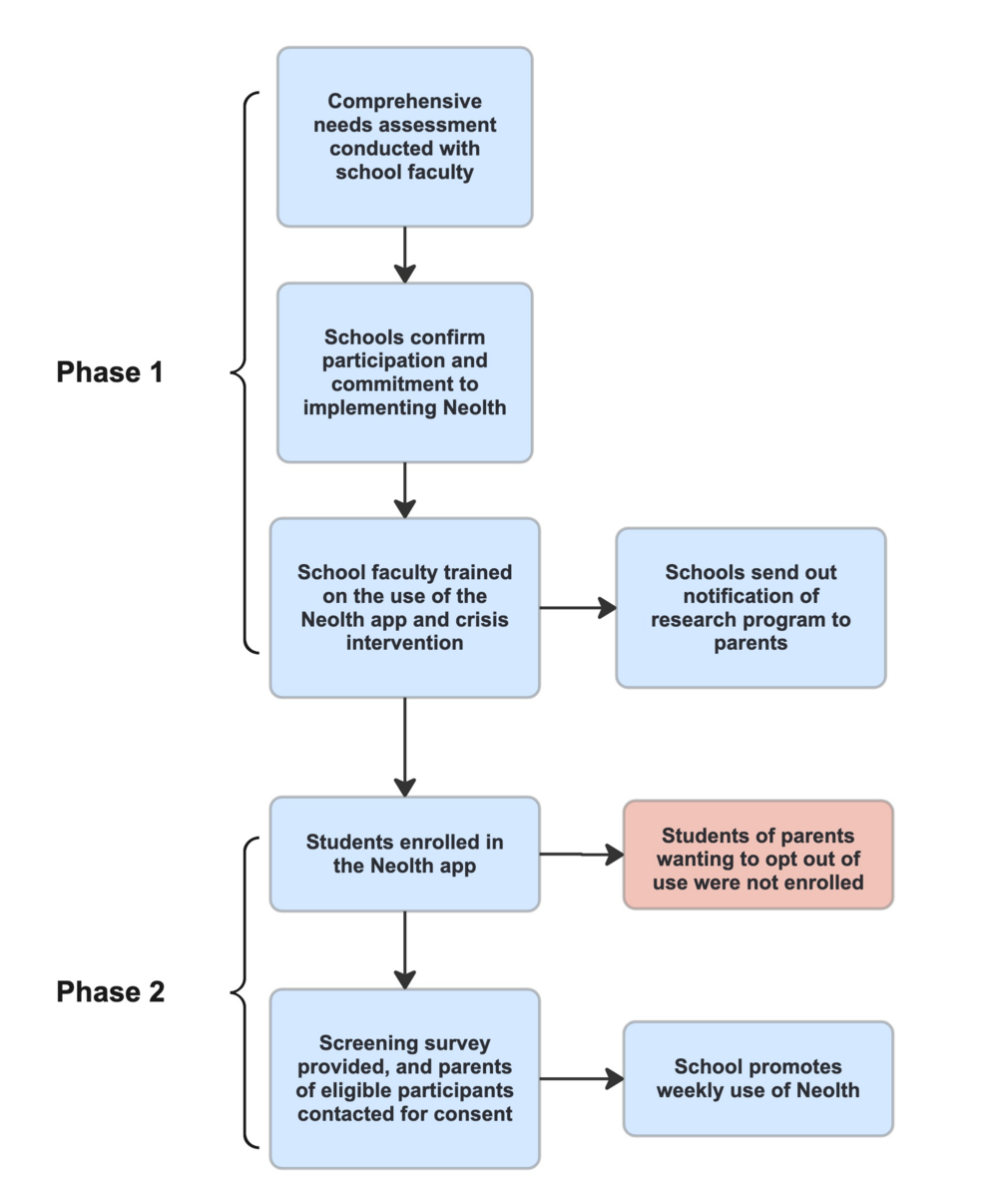
Recruitment process and school partnership methodology.

Prior to the recruitment of students, written confirmation was obtained from 3 Florida schools agreeing to participate in the implementation of the digital health solution. Designated school personnel notified all parents and guardians of students regarding the opportunity to engage with Neolth, the digital mental health solution platform. Parents and guardians receive informational materials, including a pamphlet and recruitment documentation, to facilitate the granting of consent for their child’s participation in this research initiative. Subsequently, students interested in partaking in the intervention, upon expressing their intent through opt-out forms provided by school personnel, undergo an eligibility screening process facilitated through a REDCap (Research Electronic Data Capture) link provided by study staff. Those meeting the established eligibility criteria are contacted by a dedicated research team member via telephone or Zoom (Zoom Video Communications) to seek their informed assent and proceed with enrollment in the foundational phase of the study (Figure 1).

### Ethical Considerations

The STEMSEL study adheres to stringent ethical standards to ensure the protection and privacy of all participants. This study has received ethical approval from the institutional review board at the University of Miami (UM) Miller School of Medicine (approval 20220661). The research will be conducted in accordance with the principles of the Declaration of Helsinki, ensuring the protection of participants’ rights, safety, and well-being. Written commitment letters have been obtained from the leadership of each participating school, including principals and other relevant administrators, affirming their support for the study. All procedures will be carried out with strict adherence to ethical standards, including obtaining informed consent from participants and ensuring data confidentiality. All participating schools, students, and parents or guardians were provided with detailed information about the study, including its objectives, procedures, and potential risks and benefits. Informed consent will be obtained from all participants and, for minors, from their parents or legal guardians. Each participant and their guardian will be provided with comprehensive information about the study, including its objectives, procedures, potential risks, and anticipated benefits. The consent process will ensure that participants understand their rights, including the right to withdraw from the study at any time without penalty. Data will be collected and stored securely to maintain confidentiality, with only authorized personnel having access to identifiable information. Youth and their caregivers participate in an unrecorded Zoom call with a study staff member. At the start of the Zoom call, the study staff member obtains verbal consent to send consent and assent documents via email using REDCap for an e-signature. Study staff verbally reviews approved consent and assent documents. Translation is provided for non–English-speaking parents or guardians. Caregivers and their youth sign upon agreement. The caregiver is sent a copy of the signed documents. All caregivers are asked to provide written informed consent, and all youth are asked to provide written assent digitally via email using REDCap. Caregivers and youth are provided relevant study information at the time of consent and given as much time as needed to decide on whether to participate. Written informed consent is first obtained from the caregivers or guardians of participating youth, and written assent is then obtained from youth. Caregivers and children are given a blank copy of the consent or assent form to review prior to deciding. A second copy of the consent or assent form is given to the caregiver or child after signing. Clinical site monitoring will be conducted to ensure that the rights and well-being of human participants are protected, the reported trial data are accurate, complete, and verifiable, and the conduct of the trial is in compliance with the currently approved protocol or amendments, with good clinical practices, and with applicable regulatory requirements. At UM, the data manager will manage study data under the principal investigator’s (AAS) supervision. The data manager will create computerized data collection forms to ensure the integrity and accuracy of data entry. They will help to minimize the problem of missing data by resolving omissions and errors as they arise. There will be no transfer of data from UM to Neolth. UM staff will have access to participants’ use data. Safety monitoring will include careful assessment and reporting of adverse events to the appropriate institutional review board and privacy boards. Medical monitoring will include a regular assessment of the number and type of serious adverse events. These measures ensure that the study is conducted with the utmost respect for participant confidentiality, privacy, and ethical standards.

### Data Collection

#### Baseline and Follow-Up

Participants will obtain access to the app through trained faculty on the respective campus in order to complete the preliminary baseline survey. During the preliminary baseline survey, participants will fill out a questionnaire battery that consists of standard psychological measures. Demographic information, including age, year of schooling, gender, and ethnicity will be collected. Each participant will be invited to complete the same survey a month following the completion of the intervention (at the 12-month mark) and will be monitored until 1 year later.

#### Measures

##### Overview

The study instruments are presented in Table 1.

**Table 1:** Study instruments.

Instruments	Baseline	6 Months	12 Months
Demographic survey	✓		
Perceived Stress Scale-10	✓	✓	✓
Pediatric Social Relations-Interaction With Peers-Short Form	✓	✓	✓
Patient-Reported Outcomes Measurement Information System Pediatric Family Relationships 4a Short Form	✓	✓	✓
Patient-Reported Outcomes Measurement Information System Pediatric Global Health 7+2	✓	✓	✓
National Institutes of Health Toolbox Emotional Support Short Form 8-17	✓	✓	✓

##### Perceived Stress Scale-10

The Perceived Stress Scale (PSS-10) is a 10-item questionnaire developed by Cohen et al [16], used to gauge stress levels in individuals aged 12 years and older. It examines the degree to which situations are perceived as stressful in terms of how unpredictable, uncontrollable, and overloaded respondents find their lives. Scores range from 0 to 40, with higher scores representing higher levels of stress. Average scores are calculated by adding up the scores and dividing by the number of items, providing a useful indicator for evaluating the overall level of agreement on the Likert scale (where 0=never, 1=almost never, 2=sometimes, 3=fairly often, and 4=very Often).

##### Pediatric Social Relations-Interaction With Peers-Short Form

The Pediatric Social Relations-Interaction With Peers-Short Form is an 8-item scale that monitors social relationships and peer interactions in children with neurological conditions enduring lifelong functional limitations. Each question is scored numerically based on the individual’s response (1=never, 2=almost never, 3=sometimes, 4=often, and 5=almost always), higher scores indicate better peer interactions. Scoring involves totaling the raw scores manually, followed by conversion to a *t* score.

##### Patient-Reported Outcomes Measurement Information System Pediatric Family Relationships 4a Short Form

The Patient-Reported Outcomes Measurement Information System (PROMIS) Pediatric Family Relationships 4a Short Form is a 4-item tool that assesses the determinants, outcomes, and protective impacts of children’s subjective family relationship experiences. Each question receives a numerical score based on the individual’s response (1=never, 2=rarely, 3=sometimes, 4=often, and 5=always), with higher scores indicating stronger family relationships. Scoring involves totaling the raw scores manually, followed by conversion to a *t* score.

##### PROMIS Pediatric Global Health 7+2

PROMIS Pediatric Global Health 7+2 is a 9-item scale that examines a child’s overall perception of his or her physical, mental, and social health. This scale comprises a global health score alongside 1 fatigue and 1 pain interference item, scored separately. While these 2 items are administered, they do not contribute to the global health score; instead, they provide initial estimates for pain interference and fatigue. To calculate the total raw global sum score, add the response scores of the 7 items. Refer to the raw sum to *t* score tables for the appropriate scale. For the extra 2 items in Pediatric 7+2, no sum is needed.

##### National Institutes of Health Toolbox Emotional Support Short Form 8-17

The National Institutes of Health Toolbox Emotional Support Short Form 8-17 is a self-report measure that gauges emotional support through a 7-item form for ages 8-17 years. Each item uses a 5-point scale (where 1=never, 2=rarely, 3=sometimes, 4=usually, and 5=always). The surveys are scored using item response theory methods. An item response theory–derived θ and an uncorrected standard score (*t* score) are generated for each participant. Increased scores signify greater levels of emotional support.

### Neolth

Neolth, a digital mental health app, provides preventative mental health support and education to teenagers ages 11 years and older. Neolth was devised specifically for youth as the target audience and is characteristically unique with the inclusion of school staff in the implementation of the program. Neolth’s technology creates an individualized mental health education plan for each student in order to be able to provide personalized mental health content. Their existing digital program provides stress and mental health support to teens via the self-guided platform by delivering guided activities, a practice calendar, and activity reminders. Through the program, they have readily available access to resources for relaxation, emotional awareness, health education, and stigma reduction. Over a period of 5 logins, Neolth’s evidence-based self-help process reduced teen stress [17]. In 3 weeks, Neolth reduced help-seeking stigma, acting as a stepping stone to clinical care for teens who need it [18].

Neolth is a digital app developed to serve as a resource for adolescents and young adults to support their mental health during this developmental period. It is primarily a preventive (tier 1) tool designed to support users manage mild to moderate levels of emotional distress. Additionally, Neolth offers an artificial intelligence (AI) system that flags users who exhibit signs of depression or self-harm. This feature serves as a supplementary safety measure for educational institutions, helping counselors by providing continuous monitoring of every student on a 24/7 basis. It is available for use as a mobile app, tablet, or desktop asset. The content and user interface of Neolth are based on current theories of stress and coping with the goal of maximizing accessibility and relevance while avoiding the pitfalls of a one-size-fits-all approach. Alignment with governmental support for school-based programs like SEL and the need for early identification of suicidality and distress, both optional features available to academic customers, are other priorities in the design and implementation of Neolth.

Neolth stands out as a unique digital mental health solution in several keyways. First, Neolth prioritizes school-centric accessibility, provides free access to 400 educational institutions across the United States, and ensures its widespread availability in academic settings. Second, Neolth places a strong emphasis on individualized mental health plans, as students create accounts and undergo initial assessments, forming the basis for crafting a personalized individualized mental health education plan tailored to the youth’s specific stressors and coping styles. Third, Neolth actively engages users, enabling them to shape their mental health goals and interaction preferences and fostering higher engagement and personal investment in their well-being. Fourth, the app offers tailored engagement through user-specific calendars filled with guided activities and strategic reminders via email and push notifications. Fifth, Neolth uniquely integrates students into its development, from design aesthetics free of clinical jargon to content generated by student interns and ambassadors under the guidance of mental health professionals. Finally, Neolth distinguishes itself with diverse and relevant content, addressing a wide range of mental health topics through concise videos and informative blogs, ensuring that students’ daily mental health concerns find practical solutions. Dynamic updates, including expert interviews, user-generated content, and monthly livestream sessions determined through user voting, underline Neolth’s commitment to fostering a supportive mental health community within educational settings.

### Neolth Features

Neolth offers a range of features specifically designed to address the diverse mental health needs of adolescents with precision and empathy. Recognizing that a one-size-fits-all approach is inadequate, Neolth provides personalized content recommendations that cater to individual needs rather than relying on generic anxiety or depression modules. This customization ensures that the app supports a spectrum of needs from clinical to wellness-oriented. Neolth also includes personalized crisis resources that cover a wide array of issues beyond suicide, such as domestic violence and substance use, preventing users from having to navigate extensive lists or websites for help. The community section is a valuable resource offering educational content from trusted professionals, thereby eliminating the need for adolescents to verify web-based information independently. This section also provides a private platform for learning about mental health and understanding others’ experiences. Complementing this, the Student Stories feature fosters an emotional connection by allowing users to relate to and feel supported through the experiences shared by their peers, reinforcing the sense of community and alleviating feelings of isolation.

Neolth offers a comprehensive range of features designed to support and enhance mental health and well-being. The platform includes over 100 guided practices across several categories, such as mindfulness, breathing exercises, guided imagery, creative art, meditation, journaling, and cognitive behavioral therapy. These practices address diverse needs with topics including “beat self-doubt,” “explore emotions,” “mood booster,” “rapid relaxation,” “strengthen relationships,” and “wipe out worry.” In addition to its extensive library of guided practices, Neolth offers several unique features to support users’ mental health journeys. Student Stories provide over 50 podcast and vlog-style narratives where students openly discuss their mental health experiences, fostering a sense of connection and reducing stigma. The Expert Advice Stories feature includes more than 100 interviews with health care professionals on topics such as academic stress; loneliness; health among lesbian, gay, bisexual, transgender, queer, and others; and healthy relationships with alcohol; thus offering users valuable insights and advice. To support users in monitoring their progress, Neolth includes tools for setting personalized goals, scheduling activities, tracking stress levels, and logging practice minutes, with monthly progress reports available to assess improvement. Additionally, Neolth enables users to confidentially share their progress with a therapist or doctor if desired. The platform’s integration with Apple HealthKit further enhances tracking by incorporating data on time spent engaging in mental health activities.

Teachers will be given access to Neolth’s educator platform, giving them the ability to curate lesson plans using Neolth’s content geared toward their students’ needs. Teachers can incorporate Neolth into their classroom routines in a way that suits their needs, allowing them to explore practical ways of implementing digital mental health tools in school settings. Teachers will also introduce Neolth’s mobile app to students 11+, facilitating student sign up. By using both the educator platform and student mobile app, teachers will facilitate lesson plans within the classroom and allow students to complete self-guided activities on their own time.

### Neolth Surveys

**Figure 2:**
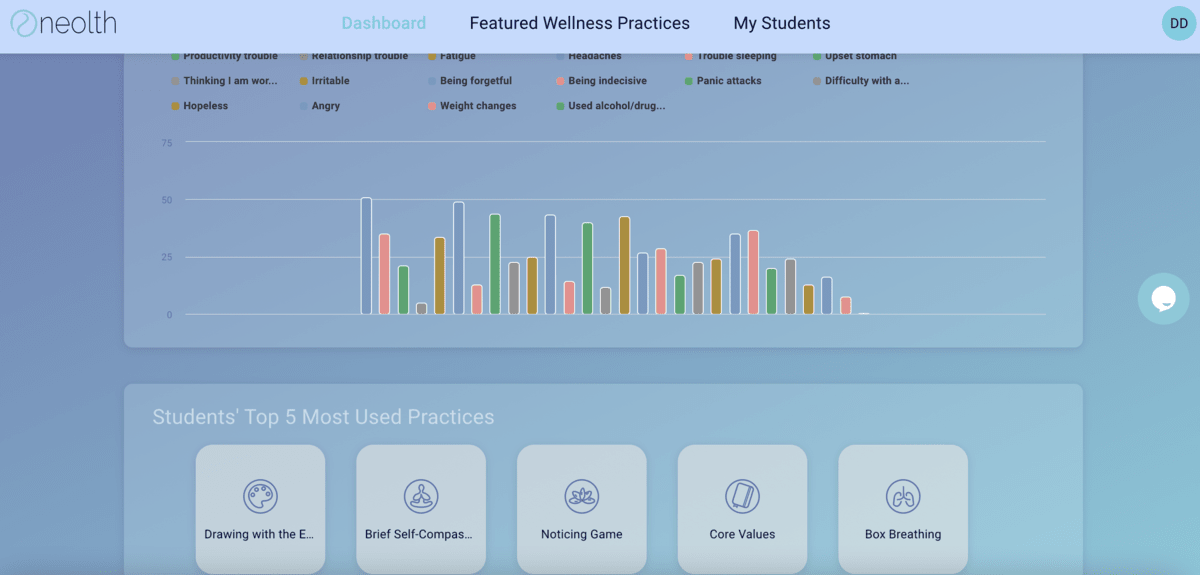
Neolth’s dashboard for schools.

**Figure 3:**
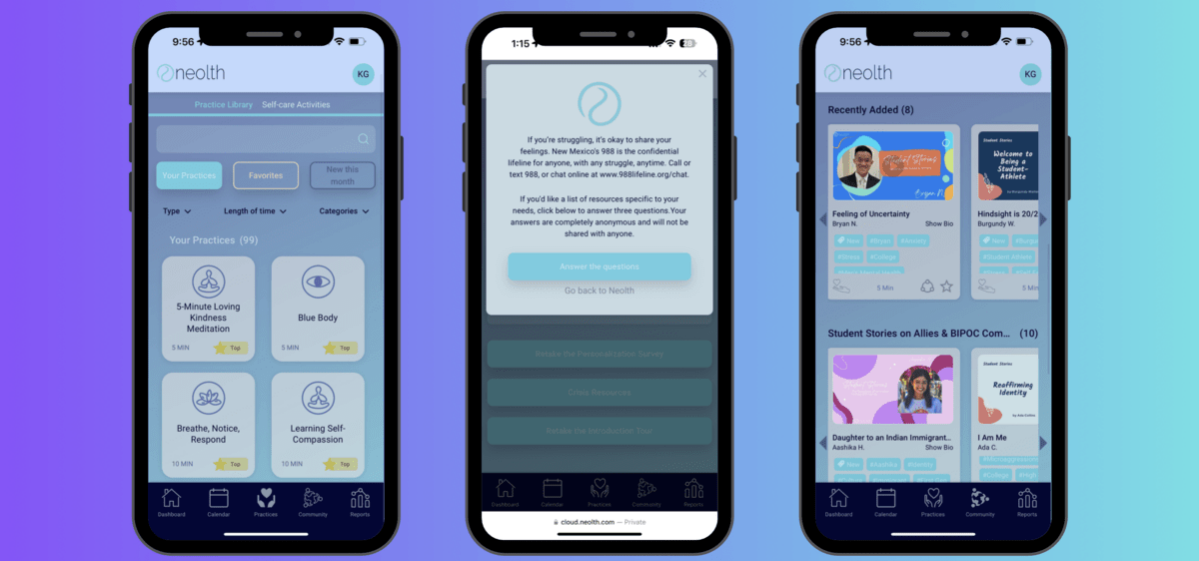
Neolth’s mobile app for students. (Explicit consent was obtained from the individuals in the image for their inclusion in the publication.).

Intake data are collected via a brief in-app survey that is presented at the time of use initiation. The survey includes questions about stress and coping, mental and physical health diagnoses, behaviors and psychoemotional symptoms, and basic demographics. Based on intake survey responses, users are presented with initial content that is tailored to their needs. As users progress through the app, additional recommended practices will become available (Figures 2 and 3).

After beginning Neolth, users will be prompted to complete weekly and monthly check-ins within the app to report on their symptoms over time. Both weekly and monthly check-ins include the Short-Form PSS-4 [19]. On the PSS-4, respondents will be asked to report on the frequency of experiencing stressful situations, rated on a 5-point Likert scale ranging from 0 to 4 (where 0=never, 1=almost never, 2=sometimes, 3=fairly often, and 4=very often). Higher total scores indicate greater perceived stress. The PSS-4 assessments will be optional, meaning users can continue using Neolth whether they complete the periodic assessments or not. Neolth compiles users’ self-assessment data recorded at check-ins within their profile, allowing users to visually track their own symptoms over time.

### App Engagement

Engagement with the app will be measured using objective session-level use data, which was provided by Neolth. Retention rates, including 30, 60, 90, and >90-day retention, were provided, as well as the average number of Neolth logins per student and the average minutes per Neolth login.

The PSS-4 is a psychological instrument used to measure the perception of stress levels. We aim to assess the impact of using the platform on users’ stress levels. The analysis of the PSS data will involve calculating mean scores and SDs to understand the average perceived stress level of Neolth users. Pre- and postuse measurements may also be compared to evaluate changes in stress levels.

The User Experience Questionnaire (UEQ) is a standardized tool designed to measure the user experience of digital products or services. It comprises various dimensions, such as attractiveness, efficiency, and dependability. By administering the UEQ to Neolth users, we aim to assess the overall experience of using the platform. The analysis will involve calculating mean scores and SDs for each dimension of the UEQ.

### Statistical Analyses

**Figure 4:**
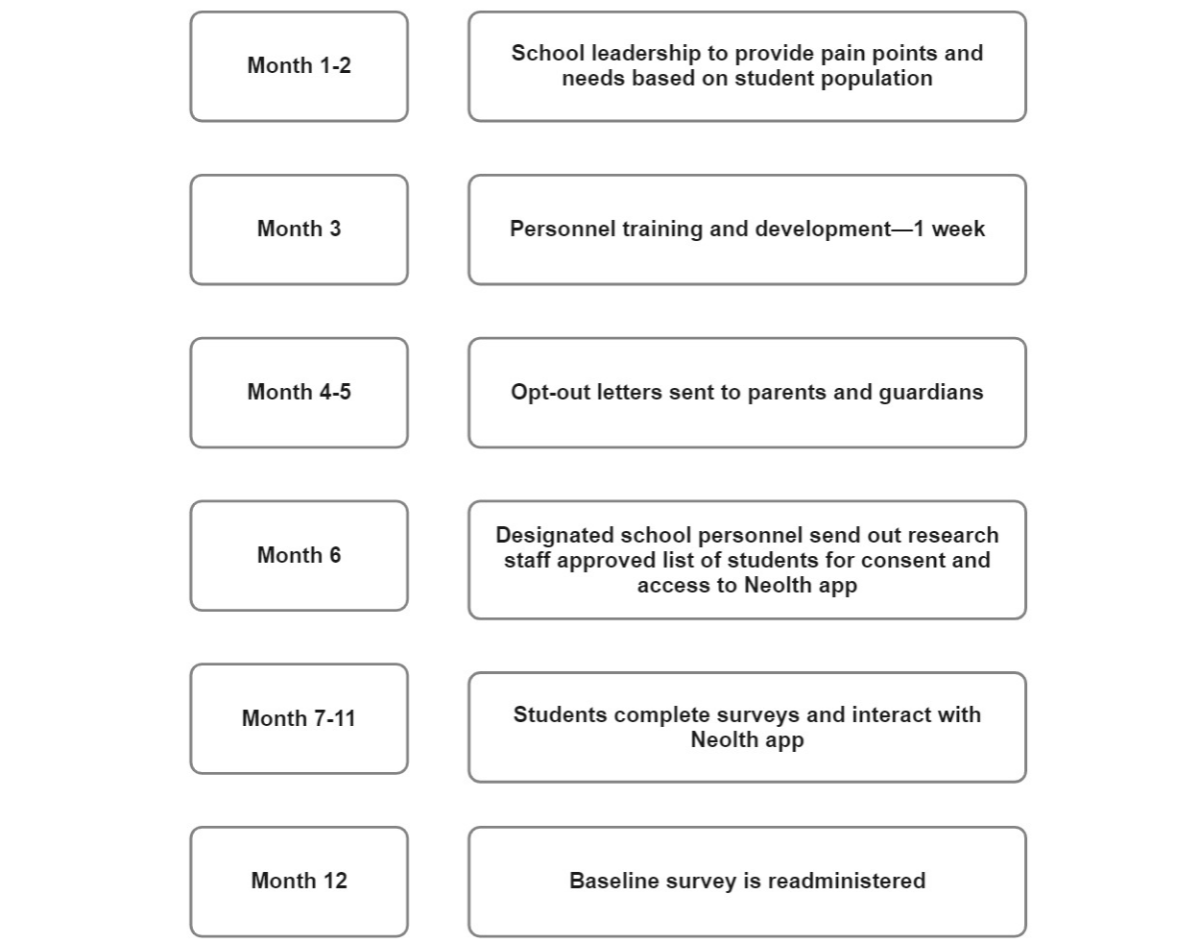
Timeline.

The statistical analyses for the STEMSEL study will be conducted using the SPSS software (version 22.0; IBM Corp). Initially, paired-sample 2-tailed *t* tests will be used to compare the mean changes in the primary outcome measures, which include students’ social and emotional well-being, anxiety, depression, and perceived stress levels, between the baseline assessment and the 12-month follow-up (Figure 4). These outcome measures will be assessed using validated instruments to ensure the reliability and validity of the results.

Subsequently, a linear regression analysis will be conducted to investigate the relationship between participants’ engagement with the Neolth platform, specifically in the context of the well-being function of the app (recorded in minutes), and changes in their mental health status. This analysis will explore whether increased engagement with the digital mental health solution predicts improvements in mental health, including reductions in anxiety, depression, and perceived stress scores. Change scores for these mental health indicators will be calculated by subtracting follow-up scores from baseline scores, with positive values indicating improvements in mental health. To account for potential confounding factors, demographic variables, such as age and gender, will be included as control variables in the regression analysis. Additionally, ethnicity, treated as multicategorical variable, will be assessed using 1-way ANOVA with the primary outcome measures. Bonferroni post hoc tests will be conducted to follow up on any significant effects, allowing for a comprehensive examination of the data. Prior to conducting the statistical analyses, relevant assumptions, including the normal distribution of outcome measures, independence, normal distribution of residuals, linearity, and homoscedasticity, will be rigorously assessed to ensure the appropriateness of the chosen statistical methods. These robust analytical techniques will provide a thorough evaluation of the STEMSEL study’s outcomes, shedding light on the impact of the digital mental health intervention on students’ mental well-being and academic performance.

Demographic information will inform our study regarding gender, age, and ethnicity background. We will calculate the mean, median, and mode to gain insight into the central tendency of the data. We will note the distribution of the data and if there is outlier information that is pertinent to the data. For the primary objective of this study, to assess whether Neolth increases social and emotional wellness in students in grades 6-12, we will perform a general linear model (GLM) approach. The GLM is a statistical approach used to analyze the relationships between the dependent and independent variables. By applying the GLM, we will be able to assess whether Neolth had a significant impact on social and emotional intelligence over time among middle and high school students.

### Crisis Escalation

**Figure 5:**
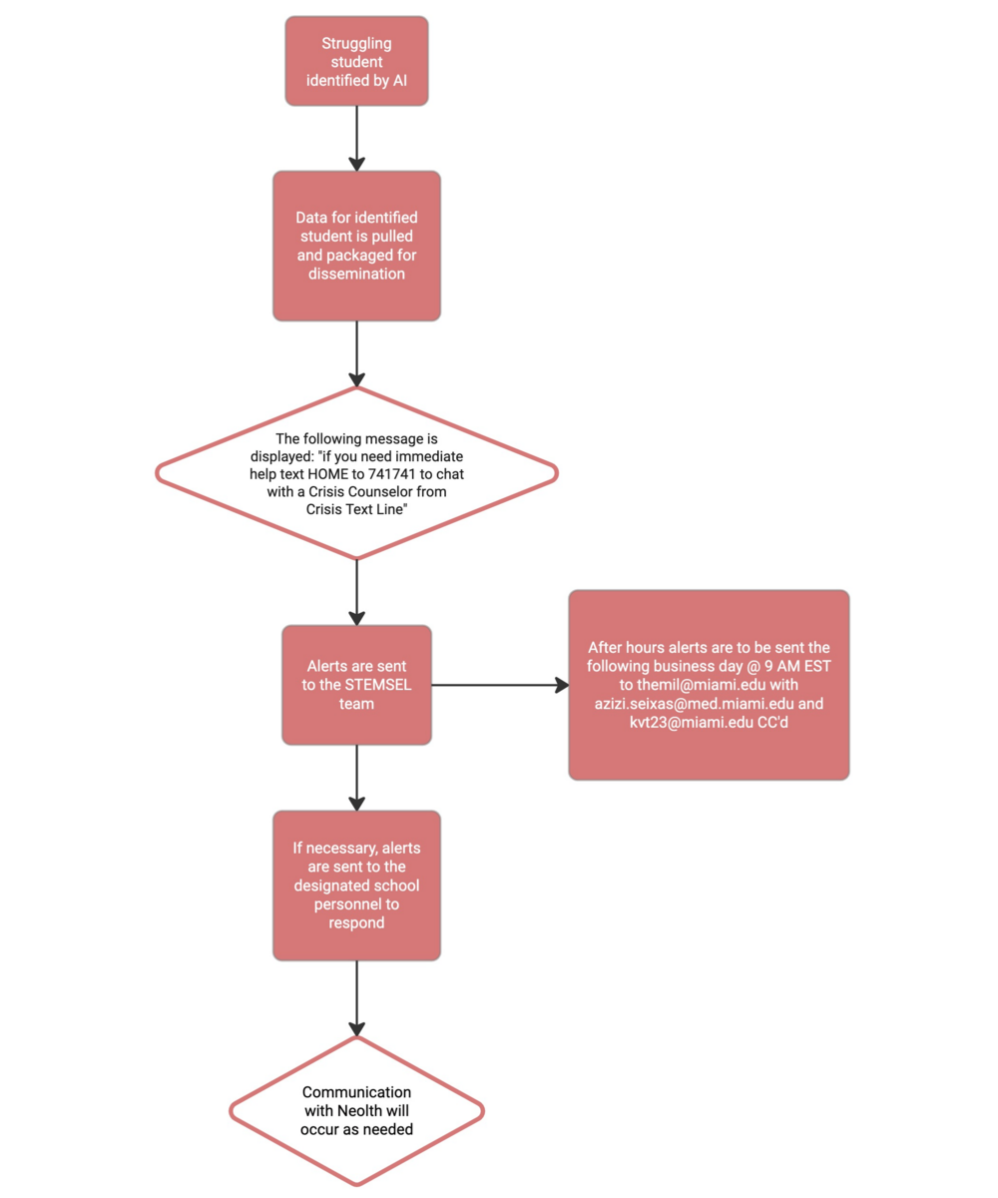
Crisis response protocol. AI: artificial intelligence; STEMSEL: Science Technology Engineering Math and Social and Emotional Learning.

As a tier 1 mental health program, Neolth offers psychoeducation, stigma reduction, and skill-building for students. However, Neolth also includes health tracking and alert features that seamlessly integrate into tier 2 and 3 supports for clinical and crisis care. Neolth’s data scientists developed an AI system that screens students on the app, flagging those who exhibit signs of depression or self-harm. This feature serves as a supplementary safety measure for educational institutions, considering that counselors are unable to provide continuous monitoring of every student on a 24/7 basis. Any data that are flagged by Neolth will be reviewed by study clinicians. If it is determined escalation is needed, school staff will be notified per their established crisis protocol so they can check in with the student. The time period from app identification to clinician review and school notification should be approximately 30 minutes, meaning school staff can intervene immediately (Figure 5).

## Results

### Phase 1 Results

Phase 1 of the STEMSEL project was dedicated to conducting a comprehensive needs assessment across the participating schools, shedding light on the barriers and facilitators for implementing the program. One prominent finding was the stark limitations in available mental health resources within the school environment. Many of the schools lacked dedicated mental health professionals, resulting in a significant shortage of support services for students grappling with emotional and psychological challenges. Additionally, more rural schools faced a shortage of community-based mental health professionals available for students to access long-term care. This resource deficit emerged as a notable barrier to providing adequate mental health care within the school setting.

Despite the resource constraints, the needs assessment revealed that many teachers and school staff exhibited a keen awareness of the mental health issues their students faced. This heightened awareness was perceived as a potential facilitator for the successful implementation of the STEMSEL program. It suggested that teachers and staff could play a pivotal role in identifying and supporting students in need of mental health interventions. Their awareness, coupled with a genuine concern for their students’ well-being, hinted at a positive synergy that could drive the program’s success. The needs assessment also highlighted that teachers and school staff exhibited a strong willingness to actively participate in the program’s training sessions. This enthusiastic response signified a high level of commitment and engagement in addressing the mental health needs of the students they served. It underscored the potential for collaborative efforts between educators and mental health professionals to create a supportive and nurturing environment for school-aged youth.

We are recruiting middle and high school students aged 11-18 years from the participating schools. The recruitment process highlights the project’s ability to engage and involve a wide range of students, recognizing that mental health challenges cut across various demographics. We are also obtaining consent from parents and legal guardians for students younger than 18 years of age, alongside securing the assent of all participating students. This phase reveals strong support from parents and legal guardians who willingly grant consent for their children’s participation. This parental support emphasizes the importance placed on addressing the mental health and well-being of the younger generation, adding a layer of validation to the STEMSEL project’s objectives.

### Implementation Lessons Learned

The initial phase of the STEMSEL project was successfully completed in August 2022 and provided valuable insights into the challenges and opportunities of program implementation. These early findings yielded several critical lessons, including the urgent need for additional mental health resources within schools to effectively support students. The project also highlighted the importance of harnessing the awareness and willingness of teachers and staff to engage in mental health initiatives. Furthermore, it underscored the significance of securing parental support and student engagement as pivotal elements for successful program implementation. These findings and lessons learned serve as the foundation for the next phases of the STEMSEL project, guiding its efforts to improve the social and emotional well-being of school-aged youth while addressing the complexities of youth mental health care in educational settings.

### Phase 2 Results

Data collection and enrollment commenced in October 2022 and is currently ongoing. Phase 2 is anticipated to conclude by the end of 2024. Data cleaning, analysis, and reporting of results are anticipated to be completed by early 2025. To date, educators at one STEMSEL site have reported positive outcomes with Neolth’s AI feature, which assesses each student’s needs and creates a personalized learning journey. Nonverbal students used Neolth’s art practices to communicate their emotions, while college-bound students benefited from Neolth’s Life Skills videos, which helped them develop communication and organization skills. The use of AI to personalize content has proven effective in addressing students’ unique mental health needs. At another study site, Neolth played a critical role in supporting a student with attendance difficulties that raised concerns about potential expulsion. Neolth’s AI algorithm identified signs of bullying and depression, prompting the school administration to address the bullying issue. The school also facilitated a connection between the student and a therapist, providing essential support. As a result of these interventions, the student is now on a path toward graduation, with noticeable improvements in their overall mental well-being and classroom engagement. This case demonstrates how Neolth’s AI-powered platform can make a significant and tailored impact on individual students, addressing their unique challenges within the educational setting.

## Discussion

### Principal Findings

The discussion on mental health in our current society has become more prevalent and has furthered the acceptance of discourse on mental health. Youth populations, however, do not have adequate resources in regard to their mental health. Digital health resources are part of a new trend for access health care. mHealth has garnered attention because of its application being either a smartphone or tablet. mHealth interventions can be used as a more effective supplement regarding mental health for the youth population compared to traditional mental health methods. In total, 89% of teens have smartphones and spend, on average, over 7 hours per day on them [20,21]. While 90% of teens have favorable opinions of mHealth apps, less than 50% feel comfortable seeking counseling from a mental health provider [18,22]. Delivering mental health support through a mobile app enables mental health professionals to reach teens who are otherwise falling through the cracks. This is especially relevant for teens of color, who are less likely to use counseling even though their risk for depression and suicide is greater [23].

The partnership with Neolth sheds light for the need of an mHealth intervention for youth-age children within the school system that is aligned with students’ generational care preferences. By providing technology-enabled support to all students, schools can normalize mental health at a young age, empowering teens with critical self-help knowledge and the courage to use clinical services when needed.

Despite these devices having a diverse selection of mental health apps, the effectiveness is still being questioned by researchers. Common themes that have created reservations about mHealth apps are privacy and confidentiality in regard to third parties having access to individuals’ data, accessibility, and equal access across all socioeconomic levels, clinical validation, and the need for ethical-legal guidance [24]. The corroboration of mHealth apps use and decreasing mental health issues in the youth population is limited. Researchers are particularly focused on the efficacy of mHealth apps because those apps are not necessarily designed with the youth population in mind. The current mHealth apps for youth are being critiqued because they are not supported by evidence-based research to back their findings and they also may not be backing evidence-based treatment guidelines for certain mental health issues in the youth population [25].

Digital mental health apps like Neolth offer significant value in addressing youth mental health issues by tackling several critical challenges. They play a pivotal role in reducing stigma by providing a private and accessible platform for mental health support, enabling users to seek help without fear of judgment. The app’s on-demand nature ensures that adolescents can access mental health resources anytime and anywhere, overcoming barriers related to geographical and scheduling constraints. By offering personalized content and treatment plans tailored to individual needs, Neolth addresses the variability in mental health challenges and preferences among youth. This personalization enhances engagement and effectiveness, as the app caters to specific issues such as anxiety, depression, and stress. To further advance this field, future research should explore how digital mental health tools can be integrated with traditional services to create a more holistic support system. Additionally, studying the long-term impacts of these apps on various demographics will provide insights into their sustained effectiveness. Enhancing user engagement and adherence through innovative features and continuous feedback mechanisms will also be essential for maximizing the benefits of digital mental health interventions.

### Limitations

This study lays out a thorough plan for implementing the Neolth intervention in 3 selected Florida schools, with a focus on its accessibility, scalability, and feasibility. Despite these strengths, several limitations must be acknowledged. The study’s restriction to 3 schools in Florida limits the generalizability of the findings. This geographic limitation means that the results may not be applicable to schools in other regions with varying demographic, socioeconomic, and educational contexts. Regional educational policies and practices unique to Florida, such as specific state mandates and funding priorities, could also influence the outcomes in ways that may not be relevant to other states. Additionally, differences in educational settings within Florida, such as urban, suburban, and rural schools, could lead to varying results not captured by the limited sample of 3 schools. Future research would benefit from involving a broader range of schools across different regions to enhance the generalizability of the findings.

The digital nature of Neolth presents potential challenges, including technical issues with the app and varying levels of digital literacy and engagement with the app outside of school hours. While Neolth’s innovative approach offers new opportunities, the intervention may not be ideal for all students, particularly those facing a digital divide or with limited access to technology. Teachers may also struggle to integrate the intervention effectively due to time constraints and competing responsibilities. Furthermore, Neolth primarily caters to English-speaking students, which may exclude non-English speakers or those with limited English proficiency. Despite these limitations, the study’s strengths lie in its potential to reach a broader audience compared to traditional mental health methods. Future studies should address these limitations by exploring diverse educational settings, overcoming digital access barriers, and ensuring inclusivity for all students to fully evaluate the efficacy and scalability of digital mental health interventions.

### Conclusions

The STEMSEL project highlights the advantages of using digital solutions to address the complex mental health and SEL needs of school-aged youth. Implementing digital interventions allows for flexible, on-demand access to resources tailored to individual needs, making it easier to reach students where they are. Engaging multiple stakeholders, including teachers, parents, students, and counselors, is crucial for overcoming the multifaceted barriers to mental health support. Schools provide a structured environment where these interventions can be seamlessly integrated into daily routines. The phased implementation and training of school staff are essential for ensuring the program’s success, as they equip educators with the skills and knowledge necessary to effectively support and sustain the intervention. This comprehensive approach aims to bridge existing resource gaps and empower educators to play a pivotal role in enhancing student well-being.

Future research should concentrate on the longitudinal effects of digital mental health interventions, particularly in understanding their long-term impact on student well-being and academic outcomes. Emphasizing the role of the decentralized trial framework, future studies need to explore how this approach can enhance the accessibility, engagement, and effectiveness of mental health tools across diverse educational settings. Evaluating how these interventions sustain their benefits over time and across different contexts will be crucial for informing future strategies and ensuring the broad applicability and success of digital mental health solutions. Finally, future studies should incorporate comprehensive training for educators and students on using digital solutions prior to deploying the app, as this will enhance digital literacy and reduce the risk of confounding variables that could affect the study’s findings.

## Abbreviations

AI: artificial intelligence
GLM: general linear model
mHealth: mobile health
MOST: moderated online social therapy
PROMIS: Patient-Reported Outcomes Measurement Information System
PSS: Perceived Stress Scale
REDCap: Research Electronic Data Capture
SEL: social and emotional learning
STEMSEL: Science Technology Engineering Math and Social and Emotional Learning
UEQ: User Experience Questionnaire
UM: University of Miami
